# Effect of artificial dermis combined with thick split-thickness skin graft composite transplantation on joint function and scar recovery in patients with joint scar contracture

**DOI:** 10.3389/fsurg.2026.1862290

**Published:** 2026-06-30

**Authors:** Huachen Wang, Yuanmei Huang, Ye Ren, Yaqing Zhou

**Affiliations:** Department of Burn Plastic Surgery and Wound Repair, The Second Affiliated Hospital of Xi’an Jiaotong University, Xi’an, Shaanxi, China

**Keywords:** artificial dermal composite grafting, generalized estimating equations, joint range of motion, scar contracture, Vancouver Scar Scale (VSS)

## Abstract

**Background:**

Joint scar contracture often leads to joint dysfunction, appearance deformity, and severe decline in patients’ quality of life. This study aimed to evaluate the effect of artificial dermal composite grafting on postoperative joint function and scar recovery in patients with scar contracture.

**Methods:**

This study retrospectively included 130 patients with joint scar contracture, who were divided into a control group (*n* = 65) and an intervention group (*n* = 65) according to the surgical method. Univariate analysis was used to compare baseline characteristics, perioperative indicators, and recontracture rates between the two groups. Three multivariate logistic regression models were constructed to analyze the effects of treatment method and baseline characteristics on the occurrence of recontracture. Generalized estimating equations (GEE) were used to analyze the effects of treatment method, follow-up time (postoperative 1 month, 3 months, and 12 months), and their interaction on active range of motion (A-ROM), passive range of motion (P-ROM), and scar score (Vancouver Scar Scale, VSS). Finally, the differences in patient satisfaction between the two groups at different postoperative follow-up time points were analyzed.

**Results:**

There were no significant differences in baseline characteristics between the control and intervention groups. The one-year postoperative recontracture rate was significantly lower in the intervention group than in the control group (6.15% vs. 32.31%, *P* < 0.001). All three multivariate models consistently identified the intervention as an independent protective factor against postoperative recontracture. GEE analysis showed that the intervention group achieved significantly greater improvements in A-ROM and P-ROM at 3 months and 12 months postoperatively compared with the control group (*P* < 0.05), and significantly better improvement in VSS at 1 month, 3 months, and 12 months postoperatively (*P* < 0.01), indicating significant improvements in scar appearance, pliability, and texture. Patient satisfaction in the intervention group was also significantly higher than that in the control group at 1 month, 3 months, and 12 months postoperatively.

**Conclusion:**

Artificial dermal composite grafting can significantly reduce the incidence of scar recontracture and markedly improve joint range of motion and scar appearance during long-term postoperative follow-up, providing a treatment option that addresses both function and aesthetics for patients with scar contracture.

## 1 Introduction

Scar contracture is a common and challenging complication that occurs during wound healing after trauma, burns, or surgery (1–3). It specifically refers to the persistent and progressive shortening and hardening of scar tissue after wound healing, leading to loss of elasticity in the surrounding skin and soft tissues and traction on the underlying joint, ultimately resulting in joint deformity, severely limited range of motion, and functional impairment (4). When the lesion involves the joint region, it often causes varying degrees of joint mobility restriction, severely affecting patients’ daily activities and social reintegration (5). Therefore, how to maximally reduce scar contracture while repairing the wound and restore joint range of motion has long been a core concern in burn, plastic, and reconstructive surgery.

Split-thickness skin graft (STSG) remains one of the important surgical approaches for treating scar contracture, particularly for repairing functional area wounds, as it allows transplantation of part dermal tissue in a single procedure (6, 7). However, its clinical application still faces several challenges: its efficacy is limited by donor site availability, and graft survival is at risk in areas with poor vascularization; more critically, the transplanted dermis may still adhere to deeper tissues and undergo scar remodeling, leading to a relatively high long-term risk of recurrent contracture, thus limiting its effectiveness in restoring long-term joint function.

In recent years, the emergence of artificial dermal substitutes has provided a new strategy for treating scar contracture (8). Artificial dermis is composed of a porous collagen–glycosaminoglycan matrix, which can promote neovascularization and fibroblast infiltration on the wound surface to form neodermis-like tissue. The subsequently overlaid thin autologous skin graft can provide effective wound coverage while retaining good elasticity and flexibility. In theory, this “artificial dermis + thin skin graft” composite transplantation approach can not only reduce the risk of scar recontracture but also improve the color, thickness, and texture of the scar.

However, most studies on artificial dermis have focused on acute burns or large-area wound repair, and evidence remains limited regarding its long-term functional efficacy in joint scar contracture surgery (9–12). Based on this, the present study focused on patients with joint scar contracture, comparing artificial dermal composite grafting with traditional STSG in terms of recontracture rate, joint range of motion (including active ROM and passive ROM), scar quality assessed by the Vancouver Scar Scale (VSS), and patient satisfaction. Using generalized estimating equations (GEE), we analyzed the interaction between intervention method and follow-up time to assess changes in various indicators at different postoperative time points, aiming to determine whether artificial dermal composite grafting provides superior long-term functional and scar-related outcomes compared with conventional STSG.

## 2 Materials and methods

### 2.1 Study subjects

This study retrospectively included 130 patients with joint scar contracture who underwent surgical treatment at our hospital from September 2022 to June 2025. Inclusion criteria were: 1) diagnosed with joint scar contracture and requiring scar release and skin repair surgery; 2) age ≥18 years; 3) meeting surgical indications and successful grafting. Exclusion criteria were: 1) concomitant severe systemic diseases (e.g., advanced cancer); 2) severe hepatic or renal dysfunction; 3) concomitant severe psychiatric disorders (major depression, bipolar disorder, schizophrenia) or poor compliance; 4) history of multiple skin grafts or other interventions at the same site resulting in severe scar destruction; 5) pregnant women; 6) missing key data (whether postoperative recontracture occurred within one year).

After applying the inclusion and exclusion criteria, patients were retrospectively grouped according to the actual surgical procedure they received in clinical practice. Patients who underwent artificial dermis combined with skin grafting were assigned to the treatment group, while those who received skin grafting alone were assigned to the control group.

### 2.2 Surgical and follow-up protocol

#### 2.2.1 Control group

After anesthesia, the patient was placed in the supine position. The wound and surrounding skin were disinfected with povidone-iodine and sterile draping was applied. The wound bed was thoroughly debrided and irrigated with diluted povidone-iodine, hydrogen peroxide, and normal saline, followed by meticulous hemostasis using a 1:100,000 epinephrine saline solution. A skin graft was harvested from the thigh or back using a dermatome according to the size of the defect. The donor site was dressed with non-adherent gauze and compressive bandaging. The skin graft was applied to the prepared wound bed with correct orientation and secured using staples or sutures, with fenestrations created when necessary for drainage. A petrolatum-impregnated gauze dressing and compressive dressing were applied, and external immobilization was used when indicated. Dressing was first changed on postoperative day 5 to assess graft take, followed by routine wound care and suture or staple removal at 12–14 days postoperatively.

#### 2.2.2 Intervention group

The procedure was divided into two phases with the following steps: Phase I Surgery (Postoperative Day 0): Debridement of burn wounds + Artificial dermal grafting + Gypsum immobilization. After successful anesthesia, the patient was placed in supine position. The wound was cleaned with soapy water, followed by routine iodine disinfection of the surgical field and sterile draping. The wound was irrigated three times alternately with iodine, hydrogen peroxide, and saline. A roller scalpel, surgical knife, and tissue scissors were used to thoroughly remove necrotic epidermis, necrotic skin, inactivated tissues, and interstitial tissue, exposing the healthy fascial layer. After confirming adequate hemostasis, 1:100,000 adrenaline saline was applied externally with precise electrocoagulation for hemostasis. The wound was then irrigated again with 3% hydrogen peroxide solution, diluted iodine solution, and saline. The wound area was measured. Artificial dermis (Lando®, BAS type) was cut according to the wound size, rinsed with saline, and prepared for use. The dermal side of the dermis was laid flat on the debrided wound with silicone membrane facing upward. The edges of the dermis were intermittently sutured to the wound margins using a stapler to ensure tight adhesion without wrinkles or dead space. The subdermal area was rinsed with saline to ensure no blood clots remained. A single-layer Vaseline gauze was applied externally, followed by compression dressing or bandage compression to ensure stable and uniform pressure. Finally, a plaster cast was applied for external immobilization.

Phase II Procedure (Approx. 14 days post-operation, awaiting vascularization of the artificial dermis): Debridement of burn wound + Autologous skin grafting + Gauze fixation. After anesthesia, the patient was placed in the supine position. The wound bed was exposed after removal of sutures and silicone layer of the artificial dermis. Necrotic tissue and excessive granulation tissue were gently debrided while preserving the underlying artificial dermal scaffold. The wound was thoroughly irrigated with povidone-iodine, hydrogen peroxide, and saline, followed by hemostasis using 1:100,000 epinephrine saline. The wound area was then measured. A split-thickness skin graft (approximately 0.25 mm) was harvested from the scalp using a dermatome after local infiltration with diluted epinephrine solution. The donor site was covered with Vaseline gauze and compressive dressing. The harvested graft was rinsed with saline and applied to the wound bed with correct orientation and secured using staples or sutures. Peripheral fenestrations were created when necessary to facilitate drainage and prevent hematoma formation. A petrolatum-impregnated gauze dressing and pressure dressing were applied, and external immobilization was used when indicated. Postoperative care included routine dressing changes, with initial assessment of graft take on postoperative day 5. Sutures were removed at approximately 12 days, followed by standard wound care and anti-scar management.

One patient in the control group developed hematoma/seroma, and one patient experienced wound infection; one patient in the treatment group developed wound infection. No severe complications such as complete graft loss or deep infection were observed.

#### 2.2.3 Rehabilitation protocol

Both groups received identical postoperative rehabilitation regimens. Starting on the first day after suture removal, patients underwent passive joint mobilization training (20 min daily, with progressive range of motion) combined with compression therapy (wearing custom elastic braces for 16 h daily) for three months. From 3 to 6 months post-surgery, the regimen transitioned to active range-of-motion exercises plus intermittent compression therapy, ensuring no significant differences in rehabilitation outcomes between groups.

### 2.3 Data collection

Baseline information collected included demographic data (age, sex, BMI, etc.) and disease-related characteristics (affected joint, duration of scar contracture, wound size, etc.). Perioperative indicators included operation time and length of hospital stay. Postoperative graft survival was recorded. Joint range of motion [Active Range of Motion [A-ROM], Passive Range of Motion [P-ROM]] and scar appearance and texture [Vancouver Scar Scale (VSS)] were collected preoperatively and at 1 month (T1), 3 months (T3), and 12 months postoperatively (T12) (13). Patient satisfaction and scar recontracture during postoperative follow-up were also collected. A-ROM was defined as the range of motion achieved by the patient using their own effort, while P-ROM was defined as the maximum joint angle achieved by the clinician without the patient's muscle strength. Due to variations in joint mobility, the range of the affected joint was expressed as a percentage of the healthy contralateral joint. Patient satisfaction was assessed using a 0–100 visual analog scale (VAS), where 0 indicated complete dissatisfaction and 100 indicated complete satisfaction. Higher scores indicated greater patient satisfaction with postoperative treatment outcomes. This scoring method has been widely used in the field of surgery to evaluate patient-reported satisfaction (14).

### 2.4 Statistical analysis

All data in this study were analyzed using R software (R 4.4.1). Normally distributed continuous variables were expressed as mean ± standard deviation (mean ± SD) and compared using independent-samples t-test; non-normally distributed data were expressed as median (range) and compared using the Mann–Whitney U test. Categorical variables were expressed as frequency and percentage, and compared using *χ*^2^ test or Fisher's exact test. Since the number of positive events (occurrence of recontracture) in the dependent variable was small, data were resampled to balance class distribution and reduce bias in multivariate models. Three multivariable logistic regression models were constructed. In Model 1, only the treatment method was included as an independent variable. Model 2 additionally included age, sex, and BMI. Model 3 further adjusted for disease-related variables, including preoperative joint range of motion (A-ROM and P-ROM), duration of scar contracture, scar area, affected joint site, and cause of injury. The dependent variable in all models was the occurrence of recontracture within one year after surgery. An odds ratio (OR) less than 1 indicates a protective factor, whereas an OR greater than 1 indicates a risk factor. Considering repeated measurements at different follow-up time points within the same patient, GEE were used for longitudinal analysis. An exchangeable correlation structure and identity link function were applied to assess the interaction between treatment method and follow-up time on postoperative joint range of motion (A-ROM and P-ROM) and VSS scores. Covariates included statistically significant variables from Model 3. Model results were evaluated using regression coefficients (*β*), standard errors (SE), and *P* values. All statistical tests were two-sided, and a *P* value < 0.05 was considered statistically significant.

## 3 Results

### 3.1 Differences in demographic characteristics and disease-related indicators between the control and intervention groups

The results showed that there were no statistically significant differences between the two groups in demographic characteristics, including age, sex, BMI, smoking history, diabetes, hypertension, and other clinical features (*P* > 0.05). The distribution of affected joints and causes of injury also showed no significant differences between the groups. Preoperative scar area, active/passive range of motion (A-ROM/P-ROM), and Vancouver Scar Scale (VSS) scores were similarly not significantly different (*P* > 0.05), indicating that the baseline characteristics of the two groups were comparable (Table 1).

**Table 1:** Baseline characteristics of the control and observation groups.

Variable	All patients (*n* = 130)	Control group (*n* = 65)	Intervention group (*n* = 65)	*P*-value
Age	47 (25–65)	47 (26–65)	49 (25–64)	0.433
Gender				0.3635156
Male	82 (63.08%)	38 (58.46%)	44 (67.69%)	
Female	48 (36.92%)	27 (41.54%)	21 (32.31%)	
BMI	25.2 (20.1–31.2)	25.2 (20.1–30.9)	25.5 (20.3–31.2)	0.255
Smoking				0.7123432
Yes	45 (34.62%)	21 (32.31%)	24 (36.92%)	
No	85 (65.38%)	44 (67.69%)	41 (63.08%)	
Diabetes mellitus				0.4370753
Yes	7 (5.38%)	5 (7.69%)	2 (3.08%)	
No	123 (94.62%)	60 (92.31%)	63 (96.92%)	
Hypertension				0.2041964
Yes	18 (13.85%)	6 (9.23%)	12 (18.46%)	
No	112 (86.15%)	59 (90.77%)	53 (81.54%)	
Joint area				
Upper limb joints				0.334958
Yes	92 (70.77%)	49 (75.38%)	43 (66.15%)	
No	38 (29.23%)	16 (24.62%)	22 (33.85%)	
Lower limb joints				0.712343
Yes	45 (34.62%)	21 (32.31%)	24 (36.92%)	
No	85 (65.38%)	44 (67.69%)	41 (63.08%)	
Joints of the head, neck, and trunk				0.528513
Yes	11 (8.46%)	7 (10.77%)	4 (6.15%)	
No	119 (91.54%)	58 (89.23%)	61 (93.85%)	
Etiology of injury				0.06205245
Thermal injury	89 (68.46%)	39 (60%)	50 (76.92%)	
Mechanical injury	36 (27.69%)	24 (36.92%)	12 (18.46%)	
Others	5 (3.85%)	2 (3.08%)	3 (4.62%)	
Time from scar contracture to surgery (months)	8 (4–12)	8 (4–12)	8 (4–12)	0.169
Scar area (cm2)	42.9 (10.8–65.0)	43.1 (10.8–65.0)	42.1 (10.8–63.9)	0.782
Preoperative active range of motion (%)	61.2 (43.3–80.0)	60.8 (44.6–80.0)	61.8 (43.3–78.9)	0.64
Preoperative passive range of motion (%)	68.1 (55.9–85.0)	66.8 (55.9–84.3)	69.4 (56.0–85.0)	0.452
Preoperative Vancouver Scar Scale (VSS)	9 (6–11)	8 (6–11)	9 (6–11)	0.117

### 3.2 Comparison of perioperative indicators and recontracture incidence between the control group and intervention group

The results showed that the median hospitalization duration in the intervention group was 11 days, significantly longer than the 9 days in the control group (*P* = 0.038). The intervention group's operative time was 76 min, significantly longer than the control group's 63 min (*P* = 0.009). The skin graft survival rate in the intervention group was significantly higher than that in the control group (*P* < 0.001). The control group had 21 cases (32.3%) of recontracture, while the intervention group had only 4 cases (6.2%) (*P* < 0.001) (Table 2). It should be noted that patients in the intervention group underwent two-stage surgery: the first stage involved debridement and artificial dermal grafting, followed by a second stage of debridement and autologous skin grafting approximately 14 days later. Theoretically, this extended hospitalization period should have been significantly longer than that of the control group receiving a single surgery. However, the observed median hospitalization difference was only 2 days, likely due to patients being discharged and recuperating between the two surgical stages, reflecting different clinical pathway arrangements.

**Table 2:** Differences in perioperative parameters and recontracture rates between the control and intervention groups.

Perioperative parameters	All patients (*n* = 130)	Control group (*n* = 65)	Intervention group (*n* = 65)	*P*-value
Length of stay (days)	10 (5–15)	9 (5–15)	11 (5–15)	0.0376
Operative time (min)	71 (48–95)	63 (48–93)	76 (48–95)	0.00858
Graft survival				0.000764996
≥90%	103 (79.23%)	43 (66.15%)	60 (92.31%)	
80%–89%	22 (16.92%)	17 (26.15%)	5 (7.69%)	
< 80%	5 (3.85%)	5 (7.69%)	0 (0%)	
Recontracture				0.000369964
Yes	25 (19.23%)	21 (32.31%)	4 (6.15%)	
No	105 (80.77%)	44 (67.69%)	61 (93.85%)	

### 3.3 Multivariate logistic regression analysis of the effects of treatment method and other baseline characteristics on recontracture

In Model 1, without adjustment for any factors, the intervention method was significantly negatively associated with recontracture (OR = 0.137, *P* = 0.001). In Model 2, adjusting for age, sex, and BMI, the intervention method still significantly reduced the risk of recontracture (OR = 0.196, *P* < 0.001). Further adjustment in Model 3 for disease-related characteristics, including scar area, joint range of motion, and affected joint, showed that the intervention method remained an independent protective factor (OR = 0.153, *P* < 0.001). Model 3 also revealed that age (OR = 1.046, *P* < 0.001) and duration of scar contracture until surgery (OR = 1.184, *P* = 0.008) were positively associated with recontracture risk, while BMI (OR = 0.913, *P* = 0.013), active range of motion (A-ROM) (OR = 0.960, *P* = 0.034), and involvement of upper limb joints (OR = 0.440, *P* = 0.008) were independent protective factors (Table 3).

**Table 3:** Multivariable logistic regression analysis of the effects of treatment method and other baseline characteristics on recontracture.

Term	Estimate	Std.error	Statistic	*p*.value	OR	CI_lower	CI_upper
Model 1							
Method	−1.985	0.580	−3.421	0.001	0.137	0.044	0.428
Model 2							
Method	−1.629	0.252	−6.469	0.000	0.196	0.120	0.321
Age	0.034	0.010	3.431	0.001	1.035	1.015	1.055
Gender	0.162	0.219	0.738	0.461	1.175	0.765	1.805
BMI	−0.077	0.033	−2.360	0.018	0.926	0.868	0.987
Model 3							
Age	0.045	0.011	4.036	0.000	1.046	1.023	1.069
BMI	−0.091	0.036	−2.488	0.013	0.913	0.850	0.981
Gender	0.139	0.241	0.575	0.565	1.149	0.716	1.844
Time.from.Scar.Contracture.to.Surgery	0.169	0.064	2.632	0.008	1.184	1.044	1.342
Scar.Size	0.009	0.021	0.415	0.678	1.009	0.968	1.051
A.ROM	−0.041	0.019	−2.121	0.034	0.960	0.924	0.997
P.ROM	−0.011	0.021	−0.511	0.609	0.990	0.950	1.030
Method	−1.875	0.288	−6.516	0.000	0.153	0.087	0.270
Joint.AreaJoints_of_the_Head__Neck__and_Trunk	0.250	0.509	0.491	0.624	1.284	0.473	3.480
Joint.AreaLower_Limb_Joints	0.066	0.313	0.211	0.833	1.068	0.579	1.971
Joint.AreaUpper_Limb_Joints	−0.820	0.309	−2.654	0.008	0.440	0.240	0.807
Etiology.of.InjuryMechanical_Injury	−0.196	0.315	−0.622	0.534	0.822	0.444	1.524
Etiology.of.InjuryOthers	−1.970	1.036	−1.901	0.057	0.140	0.018	1.063
Etiology.of.InjuryThermal_Injury	0.335	0.319	1.050	0.294	1.398	0.748	2.613

### 3.4 Generalized estimating equation analysis of the interaction between treatment method and time on A-ROM, P-ROM, and VSS

The results indicated that the intervention method had a significant positive effect on A-ROM (estimate = 1.182, *P* = 0.011) and P-ROM (estimate = 1.061, *P* = 0.018), and a significant negative effect on VSS (estimate = −1.413, *P* = 0.003). A-ROM increased significantly at 1 month (TimeT1, estimate = 13.168, *P* < 0.001) and 12 months postoperatively (TimeT12, estimate = 10.837, *P* < 0.001). P-ROM showed significant improvement at 1 month (TimeT1, estimate = 8.477, *P* < 0.001), 3 months (estimate = 4.346, *P* = 0.003), and 12 months (estimate = 12.052, *P* < 0.001). VSS decreased significantly at 3 months (TimeT3, estimate = −4.000, *P* < 0.001) and 12 months (TimeT12, estimate = −6.000, *P* < 0.001). Interaction analysis showed that the intervention group had significantly greater improvement in A-ROM at 3 months (MethodTimeT3, estimate = 4.394, *P* = 0.033) and 12 months (MethodTimeT12, estimate = 4.966, *P* = 0.011) compared with the control group; significantly greater improvement in P-ROM at 3 months (MethodTimeT3, *P* = 0.008) and 12 months (MethodTimeT12, *P* = 0.007); and significantly greater improvement in VSS at 1 month (MethodTimeT1, *P* = 0.002), 3 months (MethodTimeT3, *P* = 0.003), and 12 months (Method*TimeT12, *P* = 0.002) compared with the control group (Table 4).

**Table 4:** Effect of the interaction between treatment method and follow-up time on A-ROM, P-ROM, and VSS analyzed using GEE.

Term	Estimate	Std.error	Statistic	*p*.value
A-ROM				
Method	1.182	0.462	2.558	0.011
TimeT1	13.168	1.614	66.575	0.000
TimeT3	0.276	1.447	0.036	0.849
TimeT12	10.837	1.371	62.519	0.000
Age	0.004	0.034	0.016	0.898
BMI	0.036	0.125	0.085	0.771
Time from scar contracture to surgery	0.029	0.176	0.028	0.867
Upper limb joints	0.133	0.788	0.028	0.866
Method*TimeT1	1.236	2.277	0.294	0.587
Method*TimeT3	4.394	2.066	4.524	0.033
Method*TimeT12	4.966	1.957	6.443	0.011
P-ROM				
Method	1.061	0.448	2.368	0.018
TimeT1	8.477	1.385	37.464	0.000
TimeT3	4.346	1.450	8.987	0.003
TimeT12	12.052	1.397	74.415	0.000
Age	−0.005	0.031	0.023	0.879
BMI	−0.010	0.112	0.008	0.928
Time from scar contracture to surgery	−0.159	0.157	1.028	0.311
Upper limb joints	0.902	0.735	1.506	0.220
Method*TimeT1	2.952	1.966	1.502	0.133
Method*TimeT3	5.374	2.016	7.108	0.008
Method*TimeT12	5.256	1.950	7.264	0.007
VSS				
Method	−1.413	0.469	−3.013	0.003
TimeT1	−0.738	0.298	6.159	0.013
TimeT3	−4.000	0.219	334.509	0.000
TimeT12	−6.000	0.233	660.503	0.000
Age	0.002	0.006	0.135	0.713
BMI	0.034	0.021	2.690	0.101
Time from scar contracture to surgery	−0.043	0.029	2.156	0.142
Upper limb joints	−0.088	0.135	0.421	0.516
Method*TimeT1	−1.338	0.423	10.016	0.002
Method*TimeT3	−0.954	0.327	8.530	0.003
Method*TimeT12	−1.031	0.336	9.412	0.002

T1: at 1 month postoperatively; T3: at 3 months postoperatively; T12: at 12 months postoperatively.

### 3.5 Differences in patient satisfaction between the control and intervention groups at different postoperative follow-up time points

**Figure 1:**
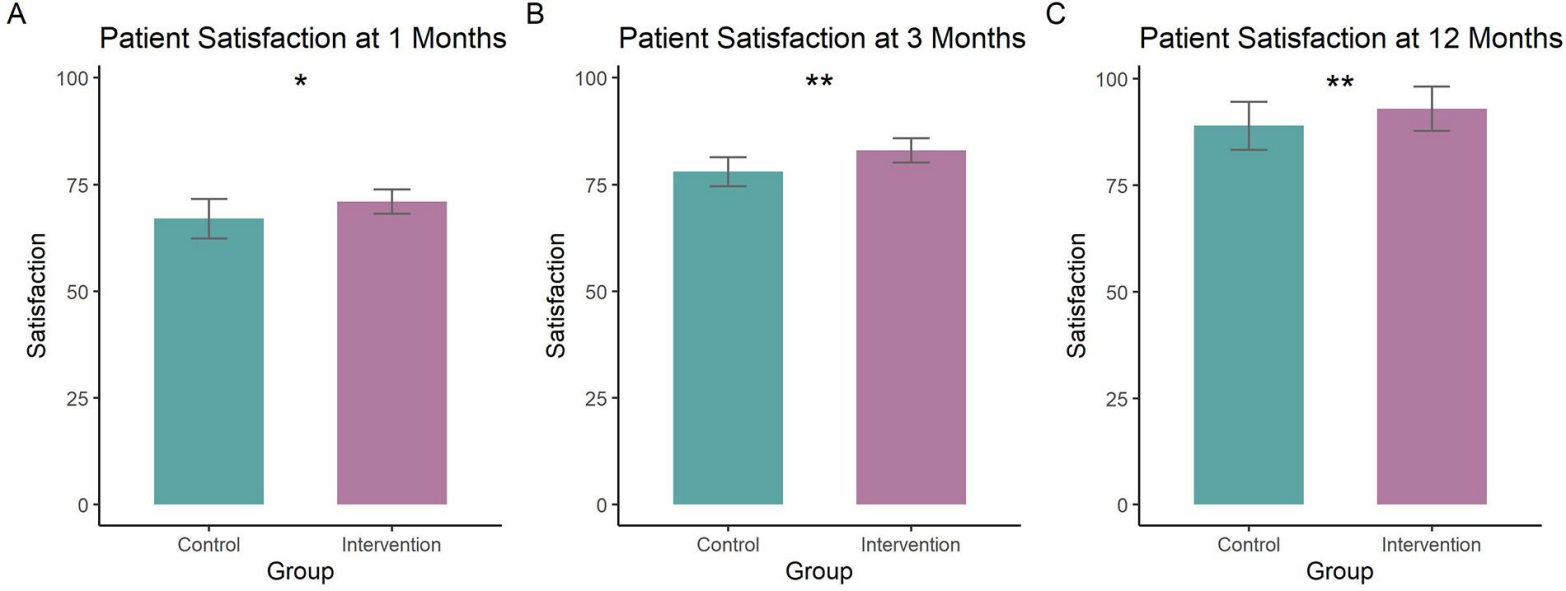
**(A)** differences in patient satisfaction between the control and intervention groups at 1 month postoperatively **(B)** differences in patient satisfaction between the control and intervention groups at 3 months postoperatively **(C)** differences in patient satisfaction between the control and intervention groups at 12 months postoperatively.

The results showed that patient satisfaction in the intervention group was significantly higher than that in the control group at 1 month, 3 months, and 12 months postoperatively (Figures 1A–C).

### 3.6 Typical patient surgical images

**Figure 2:**
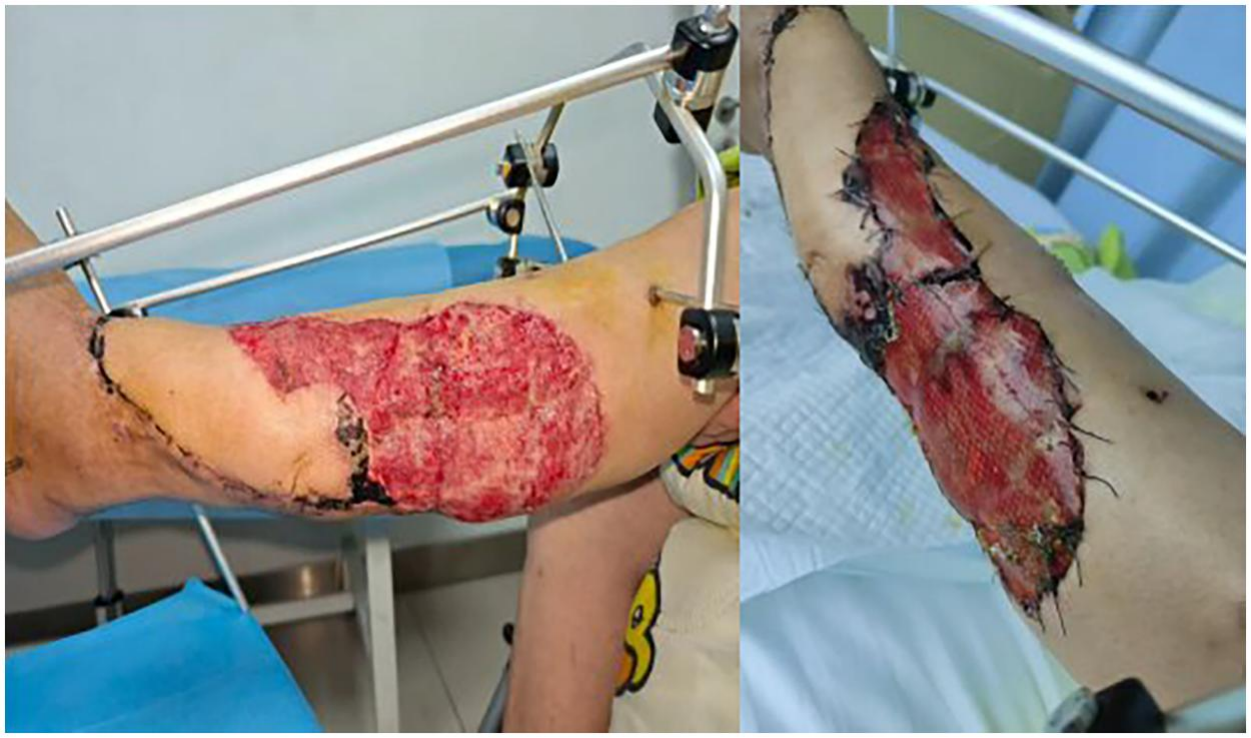
Representative images of artificial dermal composite grafting and postoperative scar recovery.

The surgical process of artificial dermal composite grafting and the postoperative recovery of typical patients further verify the efficacy of the intervention method (Figure 2).

## 4 Discussion

Our study results indicate that, compared with the control group, the intervention group significantly reduced the recontracture rate within one year postoperatively and improved graft survival. Normal scar contracture occurs because the wound lacks a dermal matrix, and the body compensates by rapidly producing large amounts of disorganized collagen fibers (mainly type III collagen). Artificial dermis provides a three-dimensional porous scaffold composed of collagen–glycosaminoglycan, structurally similar to native dermis. As fibroblasts infiltrate this scaffold, they migrate, proliferate, and align orderly along its structure rather than accumulating randomly, ultimately forming collagen fibers that are more organized and closer to normal dermis (mainly type I collagen), with improved mechanical strength and elasticity, thereby helping to reduce abnormal scar hyperplasia and contraction. Myofibroblasts, which have characteristics of smooth muscle cells, are capable of contraction and serve as the direct source of tissue contraction (15, 16). In normal wound healing, myofibroblasts undergo apoptosis at the late stage (17), but in hypertrophic scars, this process is delayed (18). An orderly dermal regenerative environment may promote timely apoptosis of myofibroblasts after they complete their function, reducing the continuous source of contractile cells. The success of STSG depends on revascularization of the dermal layer. If the dermis is thick or the wound bed has poor vascularization, dermal cells in the central region may not survive, and this area will ultimately be replaced by host fibrous tissue, forming scar rather than true functional dermis. Artificial dermis, as a scaffold, avoids necrosis and replacement issues, and its collagen alignment and elasticity can reach a more optimized state. To ensure survival, the thickness of STSG has an upper limit, which may be insufficient to fully counteract deep tissue traction when repairing severe contractures, especially around joints. The artificial dermal matrix itself has a certain thickness, and when overlaid with an autologous thin skin graft, the total thickness of the composite tissue can exceed that of most donor-site STSGs. This additional thickness provides more effective cushioning, helping to offset contractile forces to some extent and reducing the risk of recurrent scar contracture.

The interaction analysis in the generalized estimating equation indicated that the intervention group showed more significant improvement in joint range of motion during long-term postoperative follow-up. This finding has important clinical implications, as the long-term maintenance of joint mobility is a key factor affecting patients’ quality of life and functional recovery. If short-term improvement occurs but recontracture arises during long-term follow-up, patients may still face joint stiffness and limited mobility, leading to impairment in daily life and occupational function. Therefore, the sustained higher joint mobility in the intervention group during long-term follow-up further supports the advantage of this surgical approach in reducing recontracture risk and enhancing long-term functional recovery. Previous wound repair surgeries primarily focused on achieving wound closure, whereas our artificial dermis composite grafting not only provides wound coverage but also facilitates greater improvement in joint function and aesthetic appearance, breaking the vicious cycle of “injury–contracture–functional impairment” and helping to conserve healthcare resources. The improvement in VSS scores demonstrates that artificial dermis composite grafting has a clear early effect and lasting benefit in enhancing scar quality. This finding corroborates our observations of reduced recontracture rate and improved joint mobility. The potential mechanism is similar to the aforementioned analysis: the orderly environment provided by the artificial dermis fundamentally reduces inflammation and excessive collagen deposition during the scar proliferative phase, resulting in significant advantages in vascular distribution, thickness, and elasticity. At 12 months postoperatively, these advantages persist, associated with more regular collagen fiber alignment and a more complete elastin fiber network, yielding scars with color closer to normal skin and softer texture.

In addition to being related to the treatment method, joint scar recontracture is associated with age, duration of scar contracture prior to surgery, BMI, preoperative active range of motion, and the affected joint site. With increasing age, the risk of recontracture rises, which may be related to decreased skin elasticity, impaired blood supply, and reduced healing capacity in older patients, making scar tissue more prone to contracture (19, 20). The longer the contracture persists, the higher the risk of recontracture, consistent with clinical experience, as long-standing contractures lead to tissue adhesions and tendon shortening, making joint functional recovery more difficult. Moderate body weight or subcutaneous fat provides a buffering effect for the skin and scar, which is beneficial for restoring joint mobility. Higher preoperative active range of motion indicates that the joint is not severely restricted and soft tissue contracture is relatively mild, allowing greater potential for postoperative functional recovery and a lower risk of recontracture. Upper limb joints have richer surrounding muscles, tendons, and skin, good soft tissue blood supply, and patients can start functional exercises earlier, reducing stiffness and contracture (21). In contrast, lower limb joints such as the knee and ankle bear more weight during daily activities, making rehabilitation more difficult and recovery cycles longer.

This study also has certain limitations. First, as a retrospective study, there is potential selection bias in the data. In addition, the study is small in scale and single-center, and could not fully account for other potential confounding factors affecting outcomes, such as patients’ comorbidities and nutritional status. Therefore, further validation in multicenter, prospective studies is needed. Only one-year postoperative outcomes, including recontracture and range of motion, were analyzed, which may not fully reflect the overall prognosis. Future studies should include more comprehensive long-term outcome measures to better evaluate the long-term efficacy of artificial dermis combined with skin grafting in functional recovery and scar control.

## 5 Conclusion

The study demonstrates that artificial dermis composite grafting can significantly reduce the recontracture rate within one year postoperatively, significantly improve joint range of motion (A-ROM and P-ROM) at 3 and 12 months after surgery, and markedly enhance scar appearance, elasticity, and texture at 1, 3, and 12 months postoperatively. This study provides a reference for the repair of scar contractures.

## Data Availability

The original contributions presented in the study are included in the article/Supplementary Material, further inquiries can be directed to the corresponding author.
